# From Struggle to Strength: Coping with Abusive Supervision in Project Teams through Proactive Behavior and Team Building

**DOI:** 10.3390/bs14060456

**Published:** 2024-05-29

**Authors:** Qiwei Zhou, Hang Zhang, Qiong Wu, Suzana Sampaio, Anne Zouggar, Kathryn Cormican

**Affiliations:** 1 College of Management, Ocean University of China, Qingdao 266100, China; zhouqiwei@ouc.edu.cn (Q.Z.); zhanghang@nuist.edu.cn (H.Z.); 2 School of Business, Macau University of Science and Technology, Macao SAR 999078, China; 3 Department of Computer Science, Federal Rural University of Pernambuco, Recife 52171-900, Brazil; suzana.sampaio@ufrpe.br; 4 College of Science and Technology, University of Bordeaux, 33076 Bordeaux, France; anne.zouggar@u-bordeaux.fr; 5 School of Engineering, University of Galway, H91 TK33 Galway, Ireland; kathryn.cormican@nuigalway.ie

**Keywords:** abusive supervision, proactive behavior, project performance, project team creativity, team building, Proactive Motivation Theory

## Abstract

While considerable attention has been devoted to positive leadership patterns in the realm of project management, the dark side of leadership has rarely been studied within project teams. To address this gap, we focus on abusive supervision in project teams and develop a team-level moderated mediation model to examine whether, how, and when abusive supervision influences project outcomes by drawing from the Proactive Motivation Theory. Survey data were collected from 132 project teams containing 132 project managers and 392 project members using a multi-source time-lagged survey design. Our findings reveal significant negative relationships between abusive supervision and both project performance and project team creativity. Furthermore, we found that a team’s proactive behavior plays a mediating role in these relationships. More importantly, our study identifies that team building mitigates the direct negative impact of abusive supervision on proactive behavior and the indirect effects of abusive supervision on project performance and project team creativity. These findings provide valuable theoretical and managerial implications for abusive supervision and project management scholars and practitioners.

## 1. Introduction

Projects fail for several reasons, and according to Müller and Turner [1] and Sampaio et al. [2], project leadership is the primary cause of failure. The current trend of prioritizing soft skills over technical considerations has emphasized the importance of leadership in the successful implementation of projects [3,4]. This prompts researchers to explore how different types of leadership influence project outcomes. In response to these needs, project management scholars have examined different leadership styles within project-based environments. For example, Nauman et al. [4] found that transformational leadership cultivates human capital, which subsequently contributes to project success. Shared leadership, which draws upon the collective wisdom of project members, has been shown to have a substantial impact on the efficiency of project management [5]. Servant leadership can foster a collaborative culture in project teams, which in turn, enhances project performance [6]. While positive leadership styles in project contexts have received significant empirical attention, the subject of abusive supervision, which represents the dark side of leadership, remains an under-investigated area in the field of project management [7].

Abusive supervision is conceptualized as “subordinates’ perceptions of the extent to which supervisors engage in the sustained display of hostile verbal and nonverbal behaviors, excluding physical contact” [8]. Characterized as a leader’s sustained displays of nonphysical hostility and willful behavior [9], it is widely considered to be one of the most profoundly damaging leadership styles [10]. The existing literature provides ample evidence of the negative repercussions of abusive supervision (see Fischer et al. [10] for a review), and recent studies have demonstrated that abusive supervision by leaders increases subordinates’ levels of anxiety [11], reduces psychological availability [12], diminishes the passion for innovation [13], and increases the levels of employees’ intention to quit [11]. This, in turn, leads to a decline in performance, specifically work performance [14] and creative performance [15]. Although these studies have contributed to advancing the theory of abusive supervision, our study was driven by several remaining research gaps.

First, despite the recent resurgence of interest in the literature regarding abusive supervision and its harmful consequences, there is a dearth of studies investigating the concept of abusive supervision at the team level of analysis. For example, out of the 490 research studies on abusive supervision reviewed by Fischer et al. [10], only 16 of them focused on either multilevel or team-level analysis. Furthermore, there is a lack of insights available in project-based team settings. While researchers have investigated the abusive supervision theory in various team types in recent years, e.g., new venture teams [16], financial service teams [17], and R&D teams [18], studies investigating the influence of abusive supervision within a project management environment remain limited. According to Kerzner [19], project management has been conceptualized as the utilization of company resources to achieve objectives within budget and a set timeframe. Compared to work teams, project teams are characterized by transience and uncertainty, they have a predetermined end [20], and they are faced with the triple constraints of managing time, cost, and performance [21]. Thus, in such situations, project managers are inevitably subjected to mounting pressure to “do more with less”, which may make them susceptible to exhibiting abusive supervision behaviors [7]. Furthermore, scholars have advocated for further investigation into the phenomenon of abusive supervision within a wider range of team environments [22,23]. Therefore, in response to these calls and to extend the external validity of the abusive supervision theory in the project context, our first research goal is to analyze project-based teams and examine the influence of abusive supervision on project outcomes. More specifically, project performance and project team creativity were investigated. This is because project management scholars and professionals have sought solutions to achieve high levels of project performance [24,25], as well as high degrees of creativity in project teams [26,27], to enable them to maintain competitive advantages and achieve sustainability.

Second, proactive behavior, which refers to voluntary and change-oriented actions aimed at improving current circumstances [28], has been empirically proven to cultivate positive team outcomes [29]. After conducting an extensive analysis of the literature, we found that extent studies show the accumulated evidence about negative relationships between abusive supervision and proactive behavior by drawing on the Transactional Theory of Stress [28], Social Identification Theory [30], and Social Comparison Theory [31]. While these theories have provided valuable insights into understanding the link between abusive supervision and proactive behavior, their disciplinary origins impose limitations on their ability to fully elucidate how abusive supervision at the team level influences proactive behaviors within project-based settings. In this vein, we argue that the Proactive Motivation Theory proposed by Parker et al. [32] could serve as an effective theoretical lens for delineating how abusive supervision predicts proactive behavior in project team settings. This theory advocates exercising agency to actively initiate actions instead of passively observing events. The central tenet is to explain how proactive behavior is activated explicitly and comprehensively from the integrated perspectives of “can do”, “reason to”, and “energized to” motivational states. The “can do” motivational state is the belief in one’s ability to take proactive actions, the “reason to” motivational state explains why individuals or teams engage in proactive behavior, and the “energized to” motivational state involves the activation of positive emotional states, such as the drive and energy to pursue proactive goals [32]. This theory has been widely applied in illustrating the effects of transformational leadership [33,34], servant leadership [35], and supervisory support [36] on employee proactive behavior. However, it has rarely been studied to explain the association between abusive supervision and proactive behavior in teams. Therefore, our second research goal is to apply the Proactive Motivation Theory as a novel lens and uncover how abusive supervision influences proactive behavior in project teams and how proactive behavior mediates the relationship between abusive supervision and project outcomes.

Third, previous research has empirically examined various boundary conditions that serve to attenuate the negative impact of abusive supervision. For example, perceived psychological empowerment has been suggested to weaken the negative relationship between abusive supervision and employee engagement [37]; financial incentives have been proven to alleviate the negative effect of abusive supervision and ignite subordinates’ passion for inventing, which contributes to employee creativity [13]; and employment contract type has been found to moderate the abusive supervision–job performance relation, in which permanent employees are less affected by abusive supervision [38]. However, the majority of studies that investigate the buffers of abusive supervision have been conducted at the individual level, and little attention is paid to the team level. Two exceptional examples include Wang et al. [17], who demonstrated that a low intra-team competitive climate weakens the negative relationship between abusive supervision and team conflict, and Peng et al. [18], who demonstrated that the perceived frequency of change among team members strengthens the relationship between abusive supervision in teams and teams’ affective distrust. To the best of our knowledge, team building, as the core aspect of human resources management practices [3], including goal setting, role clarification, interpersonal relations and problem-solving [39], has not been theoretically proposed and empirically investigated. In order to enhance our understanding of how to reduce the negative influence of abusive supervision in project teams, our third research goal is to introduce an unexplored boundary condition, team building, and investigate its moderating effects on the relationships between abusive supervision and proactive behavior in teams, as well as project-related consequences.

**Figure 1:**
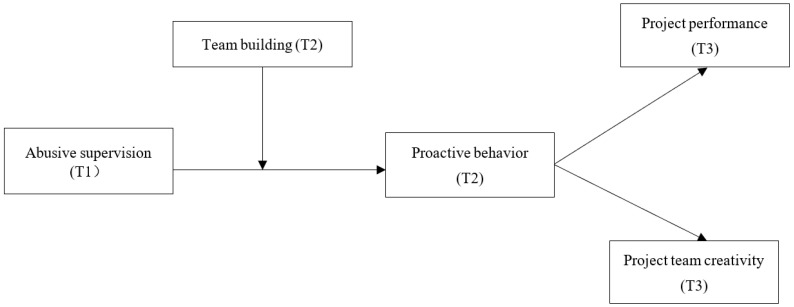
Theoretical model with hypothesized relationships among the constructs. Note: T1, T2, and T3 mean constructs were measured at Time 1, Time 2, and Time 3.

Taken together, by focusing on the project-based context, our study aims to examine whether, how, and when abusive supervision in teams influences project performance, as well as project team creativity, by introducing an internal mechanism (team proactive behavior) and a boundary condition (team building) based on the Proactive Motivation Theory. To do this, we develop a conceptual model that depicts the hypothesized relationships among the constructs in this study, as shown in Figure 1. 

By applying the abusive supervision theory to the field of project management, we believe that our study makes the following significant contributions: (1) focusing on a project-based context and conducting a team-level analysis, we fill the research gaps and extend the line of studies via examining the effects of team abusive supervision on project outcomes; (2) we examine the mediating role of team proactive behavior in the team abusive supervision–project outcomes relationship through a novel lens of the Proactive Motivation Theory; (3) we are among the first to introduce the moderating role of team building in inhibiting the negative influence of team abusive supervision; and (4) we bring insightful theoretical implications to the literature on abusive supervision and project management, as well as important practical suggestions to project managers who strive for achieving high levels of project performance and project team creativity.

## 2. Theoretical Background and Hypotheses

### 2.1. Proactive Motivation Theory

Parker et al. [32] construed a model of proactive motivation and identified proactive behavior as a goal-driven process, which comprises setting goals and striving to achieve them. Three motivational states of “can do”, “reason to”, and “energized to” are used to explain the goal-driven process. According to Parker et al. [32], the “can do” motivational state refers to the belief in one’s ability to take proactive actions, influenced by self-efficacy, control appraisals, and perceived costs. The “reason to” motivational state explains why individuals or teams engage in proactive behavior from the perspective of intrinsic motivation, integrated motivation, and identified motivation. The “energized to” motivational state involves the activation of positive emotional states that provide the necessary drive and energy to pursue proactive goals. 

Parker et al. [32] also suggested that contextual variables, such as leadership styles, can serve as distal antecedents in predicting proactive behavior by influencing team climate and fostering motivation toward proactive goals. For instance, respectful leadership can cultivate a supportive workplace climate that encourages employees to freely express themselves and take initiative without fear of negative consequences [40]. Transformational leadership, on the other hand, shapes a positive group affective tone that provides cognitive and behavioral resources necessary for maintaining goal-oriented activities and ultimately contributes to team proactivity [41]. 

Expanding upon the Proactive Motivation Theory, this study focuses on the dark side of leadership, namely, abusive supervision, and explores its influence on proactive behavior at the team level within the context of project management. Specifically, we focus on the linkage between abusive supervision and proactive behavior in teams and further examine its outcomes, specifically how and under what circumstances it happens. Furthermore, we explore how team building shapes the team’s psychological climate and patterns of interaction and, more importantly, mitigates the negative impact of abusive supervision on crucial project team outcomes, such as project performance and project team creativity.

### 2.2. Abusive Supervision, Project Performance, and Project Team Creativity

Project performance is defined as the degree to which projects achieve their stated objectives within the specified constraints of time and resources and the ability to meet or exceed stakeholder expectations [42]. Consistent with previous research findings of the detrimental effects of abusive supervision (e.g., Fischer et al. [10]; Tims and Parker [43]), this study posits that abusive supervision in teams reduces project performance. On the one hand, a common manifestation of abusive supervisory behavior is the frequent reminder of subordinates’ past mistakes and failures without acknowledging their previous achievements [8]. This pattern of constant denial and criticism tends to undermine project teams’ confidence in their abilities and diminish collective efficacy [23]. Research has shown that collective efficacy is positively associated with team performance, as it enhances the team’s ability to learn from failures [44]. Therefore, the diminished collective efficacy resulting from abusive supervision within project teams is likely to exert a negative impact on project performance. 

On the other hand, the experience of abuse and mistreatment from the team leader drives team members to question the justice of procedures, doubt whether the abusive leader values their work, and question their capacity to meet the organization’s expectations [45]. Consequently, project team members may exhibit lower levels of affective commitment that is characterized by a reduced emotional connection, identification, and involvement with the project [46]. This, in turn, leads to a reduced prioritization of tasks within the project and ultimately results in decreased project performance [46]. Therefore, consistent with much previous research demonstrating that abusive supervision is likely to reduce team performance [23,31], our research concentrates on project teams and proposes that abusive supervision in teams is negatively related to project performance.

Team creativity refers to the production of novel ideas and solutions in teams [47]. This study further suggests that abusive supervision in teams also limits team creativity, since it deteriorates the positive interactions among the project leader and project members. First, a strained relationship between an abusive leader and the abused team leads to poor quality of team member exchange. In such circumstances, the leader fails to provide interpersonal care and support to the team [48,49], which is crucial for team members to quickly adapt to the new project environment and internalize the tasks assigned by the leader [50]. As a result, the project team exhibits low intrinsic motivation and enthusiasm to engage in their work and may experience reduced satisfaction with their job. The lack of motivation and job satisfaction prohibits project team members from coming up with new ideas or adopting diverse approaches to solve problems [50]. Thus, it exerts detrimental effects on project team creativity. 

Second, when employees encounter mistreatment from an abusive leader, it fosters a negative perception of the supervisor and instills fear, which increases the likelihood of displaying aggressive and deviant behaviors, including acts of supervisor-targeted aggression [51] and job-oriented constructive deviance [52]. These behaviors can further antagonize the abusive leader, creating greater distance between them and their subordinates [53]. Consequently, this mutual alienation between the leader and members tends to result in reduced communication and information exchange within project teams. Thus, project members face challenges in receiving prompt and effective feedback from supervisors, which hinders their ability to produce valuable and innovative ideas for the project [54], which decreases project team creativity.

Based on this evidence, this study argues that abusive supervision in teams stifles both project performance and project team creativity and we therefore propose the following:

**Hypothesis** **1.** *Abusive supervision in teams is negatively related to project performance (H1a) and project team creativity (H1b).*

### 2.3. Mediating Effect of Proactive Behavior in Teams

Team proactive behavior refers to the extent to which team members proactively engage in activities aimed at improving team functioning or the team’s operating environment [55]. Drawing from the Proactive Motivation Theory [32], which involves aspiring and striving to bring about change, this study aims to delineate how abusive supervision in teams can diminish the level of proactive behavior exhibited by the team. 

First, we explain this relationship from the perspective of a “can do” motivational state, which suggests that the belief in one’s ability to take proactive actions is influenced by perceived costs [32]. Abusive supervision in teams creates an atmosphere of insecurity and stress, leaving the project team in a state of fear [56]. Abusive leaders demonstrate little tolerance for errors and failures, which fosters psychological stress and diminishes the psychological safety of the project team [57]. As a result, the abused team tends to act cautiously and carefully consider potential consequences before taking proactive actions, such as exploring new ideas or persuading leaders to change strategies. They perceive these actions as incurring additional costs, e.g., time, energy, and valuable resources, while also carrying the potential risk of failure. The perceived increase in costs deters the project team from maintaining a “can do” motivational state and results in reduced levels of team proactive behavior [32].

Second, the motivational state of “reason to” details why teams engage in proactive behavior from the perspective of motivation and can also help to explain such relationships. More specifically, abusive supervision has a detrimental impact on team members’ motivation, leaving them with little incentive to take initiative or act proactively [32]. When an abusive leader frequently emphasizes past mistakes and failures, even in instances in which considerable efforts have been invested, team members tend to experience a diminished sense of task significance [58]. They perceive tasks as unimportant, particularly in an impersonal work atmosphere in which they have little interaction with the abusive leader, which undermines their sense of engagement in meaningful work [58]. This discourages the team from striving toward proactive goals.

Third, the “energized” motivational state involves the activation of positive emotional states that generate the drive and energy to pursue proactive goals [32]. According to this perspective, emotion plays a crucial role in influencing a team’s proactive behavior. It is undeniable that perceived injustice and mistreatment from abusive leaders can evoke a range of detrimental emotions within the project team, including frustration, disappointment, anxiety, and fear [56]. Abusive supervisory practices contribute to shaping a negative affective tone within the project team, and these negative emotions are further amplified through social interactions, thereby casting a shadow on the team’s behavioral reactions [56,59]. Research has demonstrated that a positive affective tone within a team provides cognitive and behavioral resources to sustain proactive goal-regulation activities [41]. Conversely, a negative affective tone has the opposite effect, diminishing the project team’s enthusiasm and reducing their level of engagement in proactive behaviors. As a result, the project team is less inclined to take proactive actions.

Considering the abovementioned justifications, this study proposes the following:

**Hypothesis** **2.** *Abusive supervision is negatively related to proactive behavior in teams.*

Drawing upon the Proactive Motivation Theory [32], this study further postulates that the team’s proactive behavior impacted by abusive supervision would, in turn, exert influences on project performance and project team creativity.

Prior research has highlighted that a team’s proactive behavior involves both the generation and pursuit of proactive goals, which necessitates interaction with other team members, resource integration, and coordination to overcome challenges [32,60]. To some extent, it can be seen as a collaborative process of knowledge sharing and problem identification and solving, in which team members collectively find approaches to address difficulties and proactively prevent potential problems [61,62]. Accordingly, project members with less proactive behaviors would find it difficult to develop a sense of responsibility to persistently pursue team goals that have the potential to enhance project performance [63]. As a result, the reduced team proactive behavior caused by team abusive supervision is likely to diminish these collaborative efforts and ultimately lead to decreased project performance.

Furthermore, proactive behavior is characterized by its inclination toward initiating changes, suggesting that project teams demonstrating proactive behavior are motivated to initiate changes [61]. These teams have an enhanced ability to identify new information and seize potential opportunities in their environment, leading them to provide valuable suggestions for improving work procedures within the project team [61,64]. Consequently, project teams that exhibit less proactive behavior and are led by abusive leaders are prevented from generating creative ideas [64]. Furthermore, it is important to note that embarking on change initiatives does not always guarantee improvements and benefits, as there is inherent risk and the possibility of failure. Consequently, team members are concerned about being blamed for potential errors or failures, and they are discouraged from exploring new avenues and making innovative attempts. In this vein, the team’s proactive behavior suppressed by abusive supervision is likely to hinder project team creativity [43,63].

Thus, we construct the following hypothesis:

**Hypothesis** **3.** *Proactive behavior mediates the relationships between abusive supervision in teams and project performance (H3a), as well as project team creativity (H3b).*

### 2.4. Moderating Effect of Team Building

Team building is defined as a positive intervention at the team level that aims to improve social relations, clarify roles, and solve interpersonal problems that influence team functioning [39]. According to Salas et al. [65,66], team building consists of four distinct approaches, including goal setting, role clarification, interpersonal relations, and problem-solving. Goal setting refers to setting appropriate goals and planning corresponding actions to achieve them. The process emphasizes the establishment of long-term goals, planning feasible pathways, and the provision of timely rewards upon the completion of specified tasks. Role clarification facilitates employees’ comprehension of their obligations and those of the project team. It comprises the clarification of individual roles and expectations, group norms, and the shared responsibilities of team members. Interpersonal relations create a harmonious atmosphere of mutual trust by promoting support, effective communication, and the sharing of feelings. Problem-solving involves the identification and resolution of issues to enhance work-related skills [65,66]. Our study proposes that by implementing these four measures, team building can mitigate the detrimental impact of abusive supervision in teams on team proactive behavior. In addition, we utilize the Proactive Motivation Theory and its three motivational states of “can do”, “reason to”, and “energized to” to clarify this moderating effect. 

The “can do” motivational state reflects the confidence and abilities of project team members to strive for proactive goals [32]. Accordingly, we believe that the four components of team building (goal setting, role clarification, interpersonal relations, and problem-solving) diminish the role of abusive leadership and contribute to the development of proactive behavior in teams. More specifically, when team members are trained to be engaged in setting appropriate goals and formulating detailed execution plans, they experience a greater sense of control over project tasks and hold the belief that these goals are achievable [67]. Clear role clarification plays a crucial role in mitigating role ambiguity and role conflict, both of which have been identified as factors contributing to a stressful work environment. In such environments, project team members are more likely to adopt routine and well-established behavioral patterns, thereby avoiding engagement in challenging tasks and exhibiting lower levels of proactive behavior [68]. Team members who are encouraged to build good interpersonal relationships are more likely to engage in mutual trust and collaboration, which enhances their shared collective efficacy to tackle difficulties together. Furthermore, problem-solving experiences that lead to success enhance the confidence of project team members in their decision-making processes [69] and encourage more proactive behavior within teams. Taken together, effective team building equips the abused team with the capacity to recover and the confidence to believe that they can achieve project goals, which therefore diminishes the detrimental effects of team abusive supervision.

The motivational state of “reason to” provides insights into why project teams persist with specific proactive goals when faced with challenges posed by abusive supervision, such as undermining team members’ affective commitment to the organization and diminishing their sense of belonging [46]. Specifically, effective team-building practices help project members identify ways to achieve project goals [3]. When allowed to set goals for themselves, the sense of ownership from project team members is increased. They are more likely to become attentive and motivated to complete the project [32]. Furthermore, team building creates positive interpersonal relations and a conducive work environment in which project members feel supported and appreciated [70]. This generates their intrinsic motivation to actively engage in job-related behaviors that contribute to the smooth operation of the organization [32]. Therefore, our study believes that effective team-building practices facilitate the project team to conduct more team proactive behavior with enhanced motivations, which dampens the position brought by an abusive team leader.

The “energized to” motivational state describes the positive emotional states that provide the drive and energy to pursue proactive goals [32] and also helps explain the moderating role of team building. Abusive behavior in the workplace leads to negative and toxic emotions, which creates a negative affective tone within teams and has detrimental effects on team outcomes [56,71,72]. In contrast, team-building strategies foster positive team effective outcomes (e.g., mutual trust) [39] and nurture a sense of cohesion by cultivating strong interpersonal relationships between leaders and members [4,73], which could mitigate the emotional harm caused by abusive leaders within the team. Based on these observations, this study proposes that project teams, even when led by abusive leaders, may feel energized to engage in proactive behavior when team-building practices are effectively implemented. This can be attributed to the positive emotions that are activated as a result of effective team-building efforts.

Taken together, team building plays an important role in rescuing project teams under abusive supervision. It instills a sense of confidence, purpose, and proactive energy inside the team. Based on this argument, this study proposes the following:

**Hypothesis** **4.** *Team building moderates the negative relationship between abusive supervision and proactive behavior in teams, such that the relationship will be weaker when team building is higher.*

Drawing upon the Proactive Motivation Theory, we predict that abusive supervision in teams has a detrimental effect on the team’s proactive behavior, which reduces project team creativity and project performance. In addition, as a key boundary condition, team building could weaken the negative influence of abusive supervision on team proactive behavior from the “can do”, “reason to”, and “energized to” motivational states. As a result, we expect that the indirect effects of abusive supervision on both project team creativity and project performance through proactive behavior are likely to be weakened by team building. Therefore, we postulate the following integrated hypothesis:

**Hypothesis** **5.** *Team building moderates the indirect effect of abusive supervision on project performance (H5a) and project team creativity (H5b) through proactive behavior in teams, such that the mediated relationships will be weaker when team building is high.*

## 3. Method

### 3.1. Participants and Procedures

Our research participants were contacted in several ways. First, questionnaires were administered at the conference organized by the Chinese Project Management Association designed to disseminate knowledge about cutting-edge technologies, innovations, and project management practices. Second, invitations were issued to graduates of project management master’s programs from two universities in China. Third, snowball techniques were used to attract relevant participants (for instance sending emails to colleagues and collaborators who are known to be working in project-based teams and requesting them to forward our invitations to project managers or members they know).

After that, 160 project teams consisting of 528 project members and 160 project managers were invited to participate in our study. These teams operate in various industries, such as information technology, telecommunications, service, consulting, and hospitality. The inclusion of a wide range of project teams enhances the generalizability of our findings [74]. 

To mitigate the potential influence of common method variation, our data collection process was structured to occur at three distinct time points from a variety of sources, including project members and project managers [75]. Specifically, at Time 1, project members were invited to report on their typical group experience regarding the degree to which abusive behaviors are exhibited by the team leader. At Time 2, two weeks later, project members rated their experiences in team building and proactive behavior. Finally, at Time 3, two weeks later, project managers were invited to complete a survey in which they rated project performance and project team creativity.

Following the administration of the three-wave survey, a total of 28 teams were eliminated due to incomplete responses and a low within-group response rate. Our final sample contained 524 participants (including 132 project managers and 392 project members) representing 132 project teams. In terms of the demographics, among the 542 respondents, 46.3% were female, and 53.7% were male. A total of 15.1% had worked for less than 5 years, 18.7% had between 6 and 10 years of work experience, 40.6% had between 11 and 15 years of experience, 13.4% had worked between 16 and 20 years, and 12.2% had worked for more than 21 years.

### 3.2. Measures

Since all the measures were originally developed in English, we followed the standard translation and back-translation procedures [76] to create Chinese items. For all measures, a 7-point Likert scale was used, ranging from 1 = strongly disagree to 7 = strongly agree.

Abusive supervision. At Time 1, abusive supervision was assessed using the 15-item scale developed by Tepper [8]. We used a referent-shift consensus model to measure abusive supervision at the team level, in which individual team members rated survey items about their typical team experience instead of their individual experience [77]. Therefore, this scale was designed to measure the extent to which project leaders exhibit abusive behaviors while refraining from identifying project members who are subjected to such conduct. This method for assessing abusive supervision in teams has been widely used in previous research (e.g., Priesemuth et al. [23]; Rousseau and Aubé [55]). Sample items include “Our project manager gives some project members the silent treatment” and “Our project manager is rude to some team members” (Cronbach’s alpha was 0.97).

Proactive behavior. At Time 2, five items were drawn from Bateman and Crant’s [78] individual proactivity scale and modified to create team-referent items [61,79]. The items include “Members of my project team take the initiative to start new projects”, “Whenever members of my project team have a problem, they tackle it head-on”, “If members of my project team see someone in trouble, they help out in any way we can”, “Members of my project team are always looking for better ways to do things”, and “Members of my project team excel at identifying opportunities” (Cronbach’s alpha was 0.79).

Team building. Following Aga et al. [3], nine survey items across four dimensions were utilized to measure team building at Time 2. These are goal setting (example item: “Setting project goals on a participatory basis by the team”), interpersonal relations (example item: “Encouraging team members to meet with each other during the project”), role clarification (example item: “Clarifying role expectations of each team member”), and problem-solving (example item: “Involving the project team in generating ideas concerning the causes of task-related problems”) (Cronbach’s alpha was 0.83).

Project performance. We assessed project performance using twelve items derived from Dulaimi et al. [80] at Time 3. Project managers were required to assess the extent to which they believed key performance metrics were met. Sample items include “We finish projects on time”, “We finish projects within the budget”, and “Our project enables continuous improvement” (Cronbach’s alpha was 0.72).

Project team creativity. At Time 3, perceived creativity was assessed using the 8-item scale developed by Rego et al. [81] and adapted for teams by Barczak et al. [47]. Sample items include “Members of my project team suggest new ways to achieve goals or objectives” and “Members of my project team exhibit creativity on the job when given the opportunity to” (Cronbach’s alpha was 0.94).

Control variables. To address potential alternative explanations for the relationships hypothesized, we incorporated control variables that accounted for the effects of team size and gender. Variations in team size could exert effects on resources and workload requirements, which in turn, affects team performance [7]. In addition, gender diversity may play an important role in the interaction between leaders and subordinates. For example, Fritz and Van Knippenber [82] found that women receive less support from a male supervisor compared to men, whereas a female supervisor provides the same level of support to men and women. 

### 3.3. Data Analysis Technique

We employed SPSS 26 to test the hypothesized effects. To be specific, for the main effects in Hypothesis 1 and 2, hierarchical regression analyses were used. For mediation effects in Hypothesis 3, we utilized Model 4 in SPSS PROCESS v.3.5 [83]. We employed 5000-times bootstrap resamples to construct 95% bias-corrected confidence intervals. For moderation effects in Hypothesis 4, following Aiken and West’s [84] procedures, we centered team abusive supervision and team building, created their interaction term, and regressed them all on team proactive behavior. Then, we followed Aiken and West’s procedures to illustrate interactions and conducted a simple slope analysis. For moderated mediation effects in Hypothesis 5, we utilized Model 7 in SPSS PROCESS v.3.5 via 5000-times bootstrapping [83]. Project team size and the gender diversity of project members were controlled throughout the analyses. 

## 4. Results

### 4.1. Aggregating Data to the Team Level

Before conducting regression analyses, we calculated R_wg(j)_ [85] and intra-class correlations of ICC(1) and ICC(2) [86] to test whether the constructs (team abusive supervision, team proactive behavior, and team building) assessed by project members were appropriate to aggregate to the team level. The R_wg(j)_ value was used to determine the interrater agreement [85]. The intra-class correlations of ICC(1) value were calculated to examine the proportion of variance in ratings attributable to team membership, and ICC(2) was used to assess the reliability of team mean difference [86]. For team abusive supervision, the median R_wg(j)_ is 0.93, and the average R_wg(j)_ is 0.92; ICC(1) and ICC(2) are 0.30 and 0.63, respectively. For team proactive behavior, the median R_wg(j)_ is 0.88, and the average R_wg(j)_ is 0.75; ICC(1) and ICC(2) are 0.31 and 0.64, respectively. For team building, the median R_wg(j)_ is 0.89 and the average R_wg(j)_ is 0.81; ICC(1) and ICC(2) are 0.15 and 0.40, respectively. Due to the small team size (mean team size = 3.97), even though certain ICC values are not very high, it is appropriate to aggregate project team members’ responses to the team level [87] with high R_wg(j)_ [88].

### 4.2. Preliminary Analysis

We employed protocols based on good practice to validate our measurement model. The content validity of our questionnaire was ensured by using validated measures from the existing literature. The discriminant validity of the instrument was assessed by performing confirmatory factor analysis (CFA) using Mplus [89]. As the ratio of the sample size to the total number of items influences the overall model fit, we parceled the focal variables into two factors using the item-to-construct-balance approach [90] to reduce the number of parameters and improve the model fit [91]. The results show that the hypothesized five-factor model has an ideal fit with moderate modifications (χ^2^(22) = 77.84, RMSEA = 0.08, SRMR = 0.06, CFI = 0.94, TLI = 0.90). This model demonstrates a significant improvement in the chi-square value over all the alternative models, for example, a four-factor model combining team proactive behavior and team building (χ^2^(26) = 153.24, RMSEA = 0.14, SRMR = 0.12, CFI = 0.88, TLI = 0.78); a three-factor model combining team proactive behavior, team building, and project team creativity (χ^2^(29) = 219.84, RMSEA = 0.23, SRMR = 0.17, CFI = 0.81, TLI = 0.71); and a two-factor model combining team proactive behavior, team building, project performance, and project team creativity (χ^2^(31) = 253.36, RMSEA = 0.23, SRMR = 0.16, CFI = 0.78, TLI = 0.68). Thus, these focal variables were empirically distinct.

### 4.3. Correlation Analysis

Table 1 depicts the means, standard deviations [SD], correlations, and reliabilities of the variables. As shown in Table 1, the correlation between team proactive behavior and project performance (*r* = 0.40, *p* < 0.001) and project team creativity (*r* = 0.56, *p* < 0.001) are positive and significant, and the correlation between team abusive supervision and team proactive behavior is negative and significant (*r* = −0.27, *p* < 0.001), providing initial support for our hypotheses.

### 4.4. Tests of Hypotheses

Test of main effects. Hypothesis 1 posits that abusive supervision is negatively related to project performance (H1a) and project team creativity (H1b). The results presented in Table 2 indicate that the association between abusive supervision and project performance is negative and significant (*b* = −0.25, standard errors [*SE*] = 0.04, *p* < 0.001); the association between abusive supervision and project team creativity is negative and significant (*b* = −0.16, *SE* = 0.08, *p* < 0.05). Thus, Hypotheses 1a and 1b are both supported.

Hypothesis 2 argues that abusive supervision is negatively related to proactive behavior in teams. The results in Table 2 suggest that the association between abusive supervision and proactive behavior is negative and significant (*b* = −0.16, *SE* = 0.06, *p* < 0.01), supporting Hypothesis 2.

Test of mediation effects. Hypothesis 3 proposes that there is an indirect effect between team abusive supervision and project performance (H3a), as well as project team creativity (H3b) via team proactive behavior. Based on the results from PROCESS Model 4, there was a significant indirect effect between team abusive supervision and project performance through team proactive behavior (indirect effect = −0.03, *SE* = 0.02, 95% confidence interval [CI] = [−0.07, −0.01], excluding zero). Similarly, there was a significant indirect effect between team abusive supervision and project team creativity through team proactive behavior (indirect effect = −0.12, *SE* = 0.05, 95% CI = [−0.22, −0.03], excluding zero). Thus, Hypotheses 3a and 3b received support.

**Figure 2:**
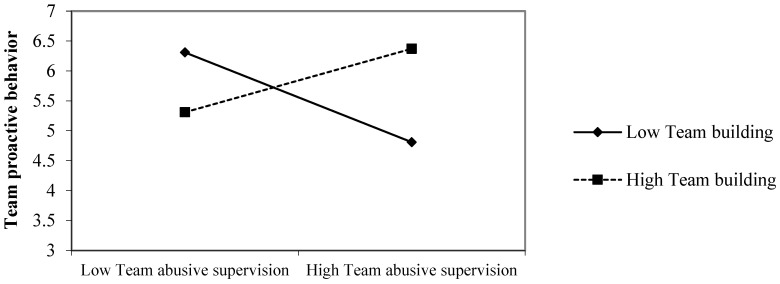
Moderating effect of team building on the relationship between abusive supervision and team proactive behavior.

Test of moderation effects. Hypothesis 4 predicts that team building moderates the negative relationship between team abusive supervision and team proactive behavior, such that the relationship will be weaker when team building is high. The results summarized in Table 3 showed that the interaction term of centered team abusive supervision and centered team building is positively related to team proactive behavior (*b* = 0.64, *SE* = 0.20, *p* < 0.01). The interaction is plotted in Figure 2. The simple slope analysis results indicated that the effect of team abusive supervision on team proactive behavior was positive but non-significant when team building was high (one *SD* above the mean, simple slope = 0.16, *SE* = 0.12, *p* > 0.05). In contrast, the effect of team abusive supervision on team proactive behavior was negative and significant when team building was low (one *SD* below the mean, simple slope = −0.36, *SE* = 0.10, *p* < 0.05). Thus, Hypothesis 4 was supported.

Test of moderated mediation effects. Hypothesis 5 probes that team building moderates the indirect effect of team abusive supervision on project performance (H5a) and project team creativity (H5b) through team proactive behavior, such that the mediated relationships will be weaker when team building is higher. As for project performance, results from PROCESS Model 7 indicated that the conditional indirect effect of team abusive supervision on project performance via team proactive behavior was positive but non-significant when team building was high (indirect effect = 0.03, *SE* = 0.03, 95% CI = [−0.01, 0.11], including zero), while it was negative and significant when team building was low (indirect effect = −0.07, *SE* = 0.03, 95% CI = [−0.13, −0.02], excluding zero). The overall moderated mediation index was significant (index = 0.12, *SE* = 0.06, 95% CI = [0.02, 0.27], excluding zero). Hence, Hypothesis 5a receives support.

A similar pattern is found for project team creativity. That is, the results of 5000 resamples of bootstrapping indicate that the conditional indirect effect of team abusive supervision on project team creativity via team proactive behavior was positive but non-significant when team building was high (*indirect effect* = 0.12, *SE* = 0.09, 95% CI = [−0.05, 0.31], including zero), while it was negative and significant when team building was low (*indirect effect* = −0.26, *SE* = 0.08, 95% *CI* = [−0.43, −0.11], excluding zero). The overall moderated mediation index was significant (*index* = 0.46, *SE* = 0.16, 95% CI = [0.18, 0.83], excluding zero). Hence, Hypothesis 5b receives support.

## 5. Discussion

By integrating the literature on abusive supervision and project management, our study aims to investigate whether, how, and when abusive supervision influences project outcomes (i.e., project performance and project team creativity). More specifically, to extend the external validity of the abusive supervision concept in the project context, our research aims to examine project-based teams and the influence of abusive supervision on project outcomes; to fully elucidate how abusive supervision at the team level influences proactive behaviors, our study applies the Proactive Motivation Theory as a novel lens and clearly explains such relationships; to enrich our understanding of how to relieve the negative impact of abusive supervision in project teams, our research analyzes an unexplored boundary condition, team building, in the relationships between abusive supervision and proactive behavior and project-related consequences. Our findings addressed our research goals by empirically demonstrating that abusive supervision has a detrimental impact on project performance and project team creativity. This is due to a decrease in proactive behavior in teams. Our study also proves that team building, a valuable and important management practice, plays a significant role in reducing the direct negative influence of abusive supervision on proactive behavior in teams. Additionally, it reduces the indirect impact of abusive supervision on project performance and project team creativity. These findings have important consequences for both theory and management.

### 5.1. Theoretical Implications

Our study makes a valuable contribution to the existing literature on project management by investigating the impact of abusive supervision on project-related outcomes. Specifically, in reviewing the literature, we note that previous studies on abusive supervision concentrate on the project-based context by examining its antecedents [7] and its individual-level consequences [5,92,93]. Our research helps to fill a gap by examining whether abusive supervision influences project outcomes at the team level of analysis. Our findings confirm that abusive supervision is significantly and negatively associated with project performance and project team creativity. This is consistent with previous research that highlights the toxicity of abusive supervision [17,18]. However, we differ from these studies by focusing on a new context, project-based teams, and our results expand the external validity of abusive supervision at the team level of analysis. We encourage future studies to extend this line of research and conduct further team-level or cross-level analysis. Additional project-related consequences also deserve further attention, and we recommend that further research examines important outcomes such as project efficiency, project effectiveness, and project sustainability.

Second, joining a handful of researchers on abusive supervision, the current study draws on the Proactive Motivation Theory and broadens our understanding of how abusive supervision influences project outcomes by introducing the mediating role of proactive behavior. Our findings are consistent with Rousseau and Aubé [55], who collected data from 82 work teams in Canadian public safety organizations and demonstrated that proactive behavior serves as a behavioral mediator in the relationship between abusive supervision and innovation in teams. In contrast, our research focuses on a different culture (Chinese). This focus helps extend the external validity and utilizes a novel lens (Proactive Motivation Theory) to advance our understanding of the relationship between abusive supervision and proactive behavior. Previous research uncovered that team-level psychological factors, e.g., collective efficacy [23], group identification [22], and team psychological safety [94], mediate the relationships between abusive supervision and team outcomes. We encourage others to extend this line of research in future studies. For example, we recommend examining mediators, such as collective psychological capital, as it deserves further attention. 

Third, while theoretically positioning and empirically offering insights into boundary conditions that influence the impact of abusive behavior, to the best of our knowledge, no studies have investigated the moderating role of team building. Our findings confirm that effective team-building strategies weaken the negative effects of abusive behavior. We integrate previous research on factors that influence proactive behavior, such as goal setting [95], role ambiguity [68], interpersonal trust [96], and problem-solving demands [97], and discovered that team building fosters a positive team climate that promotes proactive behavior. This helps protect the project team from the negative effects of abusive supervisory practices. In other words, team building serves as a buffer against the negative effects of abusive leadership on proactive behavior, which is linked to project performance and project team creativity—both of which contribute to overall project success. In summary, this study is an active response to previous calls for more studies into the team-level constructs that can mitigate the negative impact of abusive supervision in the field of project management [55].

### 5.2. Practical Implications

Our results also bring valuable practical implications for project managers and professionals.

First, the significant negative impact of abusive supervision on proactive behavior in teams, leading to reduced project performance and diminished creativity, serves as a crucial warning that highlights the necessity for project managers to eradicate all forms of abusive behaviors. To address this issue, certain measures are suggested. For instance, leadership training programs should be implemented to enhance project managers’ sensitivity toward abusive supervision and become aware of the negative effects of engaging in such behavior. Also, project managers should be urged to change their leadership style and strive to maintain long-term positive relationships with their project members, in addition to measures such as establishing zero-tolerance regulations for abusive supervision and including this as a key performance indicator for project managers. These steps would effectively prevent the occurrence of such behavior in project teams.

Second, encouraging proactive behavior in teams emerges as a valuable strategy to enhance both project performance and project team creativity. Consequently, it is crucial to guide project teams toward exhibiting proactive behaviors to foster improvements in performance and creativity. On the one hand, project managers are advised to highlight the significance of project tasks to project members and motivate them intrinsically to actively take charge. This is because motivation plays a crucial role in driving project members’ proactive engagement. On the other hand, to cultivate proactive behavior in project teams, it is recommended to organize and execute reflection team meetings [98]. These meetings provide project members with the opportunity to actively share their work, communicate, and respond to and reflect on the initiatives with each other. 

Third, our findings demonstrate that team building acts as a successful safeguard against the harmful consequences of team abusive supervision. In situations in which it may be challenging to eliminate abusive supervisory practices, it is advisable to address the issues by using effective team-building strategies as remedial measures. These include setting appropriate targets to manage expectations (goal setting), clarifying individual role responsibilities to minimize role ambiguity, enhancing project task clarity (role clarification), cultivating strong interpersonal relationships to promote trust and collaboration among project members (interpersonal relations), and offering suggestions and support in the problem-solving process (problem-solving). Notably, we acknowledge that where abusive supervision exists, it may be more difficult to implement team-building activities, and the effectiveness of the interventions may be distorted. In such circumstances, we believe that the team members may engage in and take ownership of the team-building process, and these activities might serve as an important approach for team members to address and/or cope with an abusive leader. However, this requires a more nuanced understanding of the interplay between team-building interventions and the prevailing leadership style within the team. Therefore, the potential challenges and contextual factors that influence the effectiveness of team-building activities in the face of abusive supervision warrant further examination and consideration in future research.

### 5.3. Limitations and Directions for Future Research

Several limitations should be acknowledged and should be addressed in future research. First, our study is based on survey data that do not infer causal relationships among the variables. We therefore suggest using other data collection mechanisms, such as conducting psychological experiments, to discover and further validate the causality in our proposed theoretical model. In addition, as pointed out by Barnes et al. [99], abusive supervision is dynamic. We must acknowledge that our study only captures snapshots of this process, which may underestimate (or overestimate) the consequences. We recommend that future scholars design a longitudinal research study and conduct in-depth interviews to uncover deep insights and rich information. This may enable them to draw more accurate and persuasive conclusions. Third, this study only examined proactive behavior to mediate the relationship between abusive supervision and outcomes. Previous studies pointed out that the relationships between abusive supervision and its consequences may be mediated by several factors at the same time [100]. Therefore, we advocate for future researchers to integrate multiple mediators focusing on cognition, relationships, and emotion into a single model to construct a comprehensive picture. We can then ascertain which mediators are more significant in particular contexts. Fourth, the use of a mixed methods approach to analyze team abusive supervision is also encouraged. This will provide more rigorous triangulated evidence to support our research findings. Future studies can make a valuable contribution by conducting qualitative studies, followed by surveys to test the qualitative findings, and finally use focus group approaches to develop new themes. Fifth, we acknowledge the potential drawbacks of collecting data at three distinct time points in our survey design. Several issues may cause unexpected differences in the dynamics of the project teams. For example, as time progressed, different project teams may have encountered critical incidents, such as the involvement of new clients, the replacement of team managers, or other organizational changes which may have impacted responses. Additionally, due to differences in the project schedules, even within the same time period, different project teams may have entered different stages of their project life cycle, which impacts levels of stress. Such variations could lead to unexpected differences in the interaction patterns of the project teams. To address this issue, we recommend that future researchers incorporate screening questions in the Time 2 and Time 3 surveys. For example, participants could be asked whether any critical events occurred within their project team during the previous period, and if so, they should be asked to provide details. Certain criteria could then be established to exclude teams that have undergone major influencing events. Furthermore, the appropriateness of the time interval between different survey time points should be carefully considered. In our study, we believed a two-week interval was suitable, as it captured changes in team members’ behaviors while avoiding an excessively long timeframe in which critical changes could occur. On the other hand, gathering data at three distinct time points may cause a loss of respondents in this study. For illustration, in our data collection process, the original sample at Time 1 contained 160 project teams consisting of 528 project members and 160 project managers. After one month, only 132 project teams including 392 project members and 132 project managers remained.

## 6. Conclusions

Despite the increasing recognition of project leadership’s importance in ensuring successful project delivery [3,6], we know little about the influence of abusive supervision on project outcomes. By drawing on the Proactive Motivation Theory, our study investigates how abusive supervision impacts project performance and team creativity and determines remedial measures to alleviate these negative effects. Through conducting a three-wave multi-source study involving 132 project teams, we conclude that abusive supervision inhibits proactive behavior, which in turn, leads to diminished project performance and team creativity in project teams. However, effective team-building approaches are found to weaken the negative effect of abusive supervision. Our study contributes to the literature by examining the phenomenon of team abusive supervision within the context of project management, thereby providing valuable insights and implications for both theoretical advancement and practical application in the field.

## Figures and Tables

**Table 1:** Means, standard deviations, correlations, and reliabilities of focal variables.

Variables	Mean	SD	1	2	3	4	5	6	7
1. Team gender diversity	0.53	0.33	--						
2. Team size	3.97	1.32	−0.01	--					
3. Team abusive supervision (T1)	1.84	0.56	0.31 ***	−0.35 ***	*0.97*				
4. Team proactive behavior (T2)	5.81	0.36	0.00	0.22 *	−0.27 **	*0.79*			
5. Team building (T2)	5.81	0.37	−0.53 ***	0.28 **	−0.66 ***	0.20 *	*0.83*		
6. Project performance (T3)	5.81	0.27	−0.22 *	0.28 **	−0.58 ***	0.40 ***	0.69 ***	*0.72*	
7. Project team creativity (T3)	5.54	0.52	−0.27 **	0.36 ***	−0.35 ***	0.56 ***	0.39 ***	0.52 ***	*0.94*

Note. *n* = 132. SD = standard deviation. T1 denotes that the variable was measured at Time 1; T2 denotes that the variable was measured at Time 2; T3 denotes that the variable was measured at Time 3. For gender, male = 1, female = 2. Gender diversity is based on the percentage of females in the project team. Cronbach’s alphas are presented in *italics* along the diagonal. * *p* < 0.05, ** *p* < 0.01, *** *p* < 0.001.

**Table 2:** Results for the main effects.

Variables	Team Proactive Behavior				Project Performance				Project Team Creativity			
	*b*	*SE*	*b*	*SE*	*b*	*SE*	*b*	*SE*	*b*	*SE*	*b*	*SE*
Constant	5.58 ***	0.11	5.92 ***	0.17	5.68 ***	0.08	6.22 ***	0.11	5.21 ***	0.15	5.55 ***	0.22
Controls												
Team gender diversity	0.01	0.09	0.09	0.10	−0.18 **	0.07	−0.04	0.06	−0.43 **	0.13	−0.34 *	0.13
Team size	0.06 *	0.02	0.04	0.02	0.06 **	0.02	0.02	0.02	0.14 ***	0.03	0.12 ***	0.03
Independent variable												
Team abusive supervision			−0.16 **	0.06			−0.25 ***	0.04			−0.16 *	0.08
Adjusted R^2^	0.03 *		0.08 **		0.11 ***		0.33 ***		0.19 ***		0.21 ***	

Note. *n* = 132. Statistics reported are unstandardized regression coefficients. *SE* denotes standard errors. * *p* < 0.05, ** *p* < 0.01, *** *p* < 0.001.

**Table 3:** Results for moderation effects and moderated mediation effects.

Variables	Team Proactive Behavior		Project Performance		Project Team Creativity	
	*b*	*SE*	*b*	*SE*	*b*	*SE*
Constant	5.71 ***	0.11	4.67 ***	0.31	1.22 **	0.58
Controls						
Team gender diversity	0.19	0.11	−0.06	0.06	−0.41 ***	0.11
Team size	0.02	0.02	0.01	0.02	0.09 ***	0.03
Independent variable						
Team abusive supervision	−0.11	0.07	−0.22 ***	0.04	−0.05	0.07
Mediator						
Team proactive behavior			0.19 **	0.05	0.72 ***	0.10
Moderator						
Team building	0.14	0.12				
Interactions						
Team abusive supervision × Team building	0.64 **	0.20				

Note. *n* = 132. Statistics reported are unstandardized regression coefficients. *SE* denotes standard errors. ** *p* < 0.01, *** *p* < 0.001.

## Data Availability

The data that support the findings of this study are available from the corresponding author upon reasonable request.
